# Investigating Cellular Trajectories in the Severity of COVID-19 and Their Transcriptional Programs Using Machine Learning Approaches

**DOI:** 10.3390/genes12050635

**Published:** 2021-04-24

**Authors:** Hyun-Hwan Jeong, Johnathan Jia, Yulin Dai, Lukas M. Simon, Zhongming Zhao

**Affiliations:** 1 Center for Precision Health, School of Biomedical Informatics, The University of Texas Health Science Center at Houston, Houston, TX 77030, USA; hyun-hwan.jeong@uth.tmc.edu (H.-H.J.); johnathan.jia@uth.tmc.edu(J.J.); yulin.dai@uth.tmc.edu (Y.D.); lukas.simon@bcm.edu (L.M.S.); 2 MD Anderson Cancer Center UTHealth Graduate School of Biomedical Sciences, Houston, TX 77030, USA; 3 Therapeutic Innovation Center, Baylor College of Medicine, Houston, TX 77030, USA; 4 Human Genetics Center, School of Public Health, The University of Texas Health Science Center at Houston, Houston, TX 77030, USA

**Keywords:** COVID-19, bronchoalveolar lavage fluid, single cell RNA-seq, trajectory inference, machine learning, deep learning

## Abstract

Single-cell RNA sequencing of the bronchoalveolar lavage fluid (BALF) samples from COVID-19 patients has enabled us to examine gene expression changes of human tissue in response to the SARS-CoV-2 virus infection. However, the underlying mechanisms of COVID-19 pathogenesis at single-cell resolution, its transcriptional drivers, and dynamics require further investigation. In this study, we applied machine learning algorithms to infer the trajectories of cellular changes and identify their transcriptional programs. Our study generated cellular trajectories that show the COVID-19 pathogenesis of healthy-to-moderate and healthy-to-severe on macrophages and T cells, and we observed more diverse trajectories in macrophages compared to T cells. Furthermore, our deep-learning algorithm DrivAER identified several pathways (e.g., xenobiotic pathway and complement pathway) and transcription factors (e.g., MITF and GATA3) that could be potential drivers of the transcriptomic changes for COVID-19 pathogenesis and the markers of the COVID-19 severity. Moreover, macrophages-related functions corresponded more to the disease severity compared to T cells-related functions. Our findings more proficiently dissected the transcriptomic changes leading to the severity of a COVID-19 infection.

## 1. Introduction

The novel SARS-CoV-2 virus has caused a total of 22 million COVID-19 cases and nearly 380,000 deaths in the United States since 21 January 2020 [1]. COVID-19 patient symptoms exhibit significant variation, ranging from being asymptomatic to death [2]. COVID-19 infection mortality risk factors such as age, smoking status, gender, diabetes, and hypertension have been identified [3]. While the majority of patients experience no symptoms to moderate symptoms such as loss of taste or smell, fever, and chills, some patients develop respiratory failure and require hospitalization. Furthermore, there are patients that develop a severe infection despite having no known risk factors at all [4]. In addition to patients developing clinical psychological sequelae such as post-traumatic stress disorder, depression, and anxiety after a COVID-19 infection, the unpredictability of these infections continues to drive the worsening mental health of the general public [5]. 

As the number of cases and fatalities continue to rise, identifying the key mechanisms that modulate the severity of an infection is essential. There is growing evidence that immunopathology may play a significant role in the development of severe clinical sequelae in infected patients [6]. Patients may develop cytokine storms resulting in various symptoms such as disseminated intravascular coagulation, respiratory failure, and shock [7].

Single cell RNA sequencing (scRNA-seq) is a high-throughput technique that enables the examination of gene expression of the cellular heterogeneity at an individual cell level [8]. It has been applied to COVID-19 studies to understand the mechanisms of disease, although such data is currently very limited due to the unavailability of human tissues from the patients. Liao et al. generated and analyzed bronchoalveolar lavage fluid (BALF) scRNA-seq data to reveal the landscape of bronchoalveolar immune cells in COVID-19 patients [9]. They collected 66,452 cells in total with 13 samples, and the study identified the presence of proinflammatory monocyte-derived macrophages in severe patients and CD8+ T cells in moderate cases. Several groups followed up with additional analyses using other data. For example, Liu et al. reanalyzed the same data in addition to bulk-tissue RNA-seq data. They detected SARS-CoV-2 gene expression in as many as eight immune-cell types including macrophages, CD8+ T cells, and NK cells [10]. Moreover, they identified an abundance of ORF10 and a high ORF10/N ratio in severe cases, which had not been previously reported [10]. Xu et al. performed another reanalysis of Liao et al.’s data by integrating the data with peripheral blood mononuclear cell scRNA-seq data [11]. They reported anomalous activation of BALF monocyte-macrophages in severe COVID-19. Although the previous work identified cellular features that are differentiated by the disease severity, it is still unknown what are the cellular trajectories of COVID-19 disease progression, i.e., healthy-to-moderate and healthy-to-severe changes, as well as what cellular features drive differentiation of the pathogenesis. In addition, as SARS-CoV-2 continues to rapidly evolve, identifying driver genes and transcriptional programs that respond to an infection will be useful for predicting the near future trend of the pandemic [12,13]. 

In this study, we hypothesize that there are linear cellular trajectories and transcriptional programs involved in the pathogenesis of COVID-19, which could be inferred from COVID-19 BALF scRNA-seq data. We further hypothesize that there are different cellular trajectories according to cell types and disease severity, and there are transcriptional programs that differentiate the cellular trajectories. To this end, we applied novel bioinformatics and machine learning methods to the Liao et al. scRNA-seq data to uncover the underlying biological mechanisms of the COVID-19 pathogenesis at single-cell resolution. We first applied the Slingshot algorithm [14] to infer the cellular trajectory from healthy-to-disease (moderate or severe) patients in two cell types: T cells and macrophages, both of which were found correlations between their abundance and COVID-19 severity in Liao et al. [9]. Other cell types were not used due to an insufficient number of cells. We subsequently assessed the biological pathways and transcriptional programs in each cell type using DrivAER (Driving transcriptional programs based on AutoEncoder derived Relevance scores), a deep learning algorithm developed in our lab [15]. These findings provided some important insights into the cellular changes during the infection and toward patient severity. 

## 2. Materials and Methods

### 2.1. COVID-19 BALF scRNA-Seq Data

We retrieved COVID-19 BALF scRNA-seq data of 13 patients (severe (n=6), moderate (n=3), and healthy (n=4) cases) generated by Liao et al. [9] from the UCSC Cell Browser [16]. The severity of infection was defined by the patient’s symptoms. Severe COVID-19 infection was defined as requiring ventilation support and/or the presence of pneumonia in the lungs as opposed to the moderate patients, which only exhibited symptoms such as fever, chills, and nausea [9]. More detailed information is available in Liao et al.’s publication. We retained 49,417 macrophage cells and 7716 T cells based on the scRNA-seq data and annotations provided by the original paper. We then performed data filtration, normalization, dimensional reduction (Principal Component Analysis (PCA) and Uniform Manifold Approximation and Projection (UMAP) [17]), data integration, and clustering using Seurat version 3.2.3 for each cell type [18]. 

We processed Liao et al.’s data as follows: First, we identified 2000 highly variable genes per sample using the FindVariableFeatures function in Seurat. Second, with the identified highly variable genes, we performed canonical correlation analysis for data integration using the IntegrateData function implemented in Seurat [18]. Next, we normalized and integrated the gene-expression data using the NormalizedData and ScaleData functions in Seurat. Afterward, we performed PCA to calculate 30 principal components (PCs) of the integrated data via RunPCA and RunUMAP functions in Seurat. The first 20 PCs were used for UMAP embedding and clustering. At the end of the process, cell clustering was performed with the FindNeighbors and FindClusters functions. As a result, the process generated three types of outputs: a pre-processed gene-expression matrix, a cluster label of each cell, and cell embeddings. The cluster information and embeddings were used in the trajectory inference step, and the pre-processed gene-expression matrix was used during the DrivAER analysis.

### 2.2. Trajectory Inference Using Slingshot

Differentiation of the pseudotime of a lineage between sample groups could indicate a transition of the disease status (e.g. healthy-to-severe), as demonstrated in Fu et al. [19]. Slingshot is a top-performer for cellular trajectory from single-cell RNA-seq data, according to the recent benchmarking by Saelens et al. [20]. Saelens et al. also suggested using it to infer a linear trajectory (i.e., a disease progression), which suits our assumptions. We used Slingshot to infer cellular trajectories (i.e., pseudotime of cells) between two groups, and the following paired disease transitions were considered: healthy-to-moderate (H→M) and healthy-to-severe (H→S). Slingshot requires a dimensional-reduced cell matrix and cluster labels of each cell as input, which we generated by using Seurat. For each trajectory inference, we set the parameters start.clus (the cluster containing the starting point of the trajectory) and end.clus (the cluster with the ending point of the trajectory) to infer the expected cellular trajectory (i.e. from the first group to the second group) based on the cell population of each group. As a result, slingshot generated multiple lineages. A single lineage represented a path in a cellular trajectory (i.e., possible COVID-19 pathogenesis on the cell type) and the cells belonging to the lineage were ordered by pseudotime calculated by slingshot. Since the next step (i.e., transcriptional program analysis) required us to select a lineage, we empirically selected a lineage based on the following criteria: (1) the number of cells covered by the lineage, preferably more cells, and (2) pseudotime distribution between the two groups, preferably a lineage with a higher distribution of the disease group (moderate/severe). Wilcox signed-rank test and fold-change calculation of pseudotimes between two groups were performed to evaluate the second criterion.

### 2.3. Transcriptional Program Identification

In this study, we applied DrivAER, a deep-learning based algorithm to identify transcriptional programs that potentially regulate the inferred cellular trajectories [15]. DrivAER is a transcriptional program identification method that utilizes deep learning and machine learning techniques. Transcriptional programs are sets of genes that are co-regulated (e.g., targets of a transcription factor (TF)) or have common biological functions (e.g., genes in a pathway), and their functions potentially determine a cellular response such as cellular trajectory or condition. A previous study demonstrated that DrivAER accurately identifies transcriptional programs driving cellular response [21].

To test whether a transcriptional program is a potential regulator of the cellular response, DrivAER works as follows: First, DrivAER reduces the gene expressions of the transcriptional program into a low dimensional data manifold using the deep count autoencoder (DCA) [22]. Secondly, DrivAER uses random forest models to calculate a relevance score quantifying the association between the cellular manifold coordinates and their cellular response. If the cell responses are continuous like pseudotime values, $R^{2}$, the coefficient of determination between prediction by a random forest and the observed cellular response, will be calculated as a relevance score. A higher relevance score indicates the transcriptional program potentially regulates the cellular response and vice versa. Transcriptional program annotations from MSigDB [23] and TRRUST [24] were collected and used in this study. 

Since we have two different trajectories for each cell type (H→S and H→M), we designed $\;\Delta \;RS=RS_{H\to S}-RS_{H\to M}$, where $RS_{H\to S}$ and $RS_{H\to M\;}$ represent relevance scores of a transcriptional program in H→S and H→M trajectories, respectively. ΔRS scores the differentiation of a transcriptional program between two conditions. When ΔRS is greater than 0, it indicates that the transcriptional program may drive COVID-19 infections towards a severe state, and ΔRS < 0 indicates that the role of the transcriptional program may stabilize the patient’s infection severity. When ΔRS is close to or equal to 0, it means there is no differentiation between two trajectories.

## 3. Results

### 3.1. Macrophages and T Cells Exhibited More Diverse Cellular Trajectories from Healthy Controls to COVID-19 Patients

**Figure 1:**
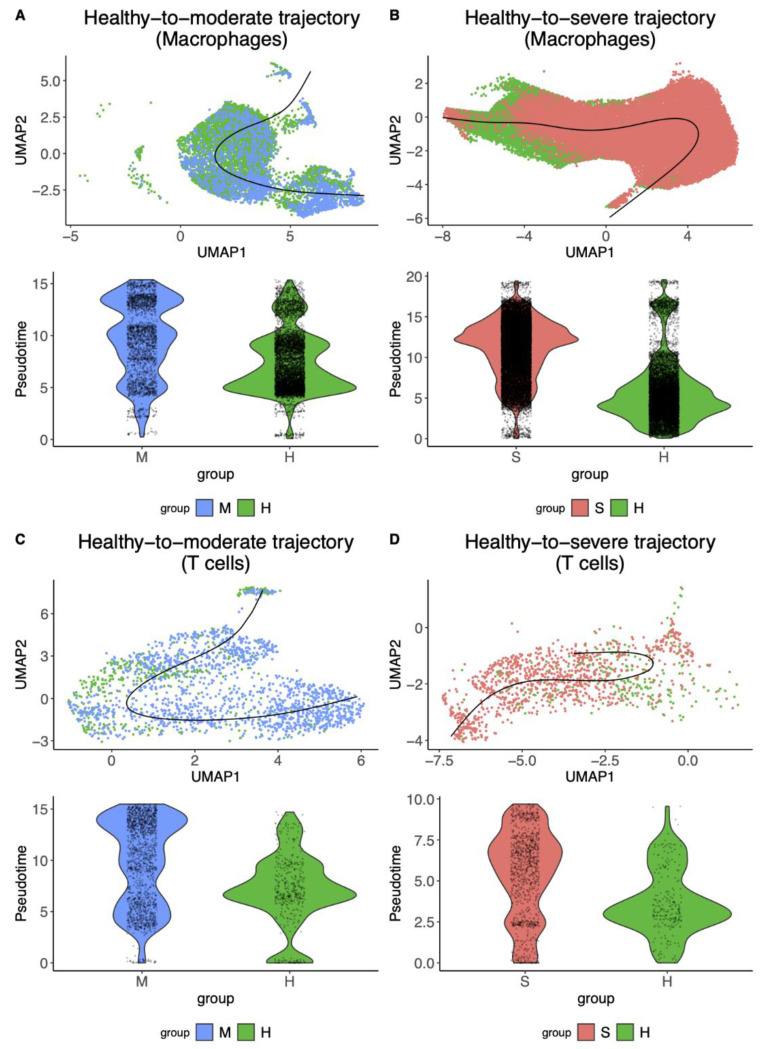
Cellular trajectories inferred by Slingshot in macrophages and T cells using Liao et al.’s BALF scRNA-seq data [9]. The illustrations of UMAP embedded cells (colored by the disease status) and inferred cellular trajectories (displayed as bold lines) (top). Violin plots show the pseudo time distributions of the inferred cellular trajectories for each disease group (healthy vs. moderate/severe) (bottom). (**A**,**B**) The cellular trajectories from healthy control cells to moderate or severe cells in macrophages. (**C**,**D**) The cellular trajectories from healthy control cells to moderate or severe cells in T cells. UMAP: Uniform Manifold Approximation and Projection. H, M, and S denote healthy, moderate, and severe samples, respectively.

We first performed trajectory inference for four cases: H→M and H→S on macrophages and H→M and H→S T cells using the Slingshot algorithm (Figure 1, Table 1, Supplementary Figure S1, and Supplementary Table S1). Slingshot detected cellular trajectories with multiple lineages for every case. We observed that the H→S of both macrophages and T cells showed more diverse cellular trajectories that could differentiate the disease status (six and four lineages), while the H→M only revealed a smaller number of the differentiated lineages (three and one linages). Next, we selected the top lineage of each transition and found that H→S of macrophages showed better differentiation than T cells (0.94 fold-change vs. 0.56 fold-change respectively, Figure 1A,C), and H→M of T cells showed better differentiation than macrophages (0.47 fold-change vs. 0.37 fold-change respectively, Figure 1B,D). This result suggested that studying macrophages would be informative to understand what cellular features/mechanisms aggravate the COVID-19 symptoms, and T cells would be helpful to study how the immune system reduces COVID-19 infection severity.

### 3.2. SARS-CoV-2 Gene-Expression Pattern Could Dissect the Healthy-to-Severe Trajectories into Three Different Stages of COVID-19

To investigate how cellular trajectories correlated with the SARS-CoV-2 virus, we explored the patterns of SARS-CoV-2 gene expression, which was quantified in Liu et al. [10], across the pseudotime in the H→S trajectories for macrophages and T cells (Supplementary Figure S2). We were unable to perform the analysis for the H→M  trajectories because only a small number of cells contained SARS-CoV-2 gene transcripts in the moderate cell population [10]. We observed most of the gene expressions were located in the middle stage for both trajectories of macrophages and T cells (Table 2), suggesting that the inferred healthy-to-severe pathogenesis at the single cell level consists of three stages: (1) cells without infection, (2) cells with SARS-CoV-2 virus infection, and (3) development of the disease and symptoms. Moreover, we found an enrichment of the ORF7a gene-expression at the late stage in macrophages (Supplementary Figure S2). We assumed that ORF7a expression at the late stage might indicate macrophages’ ingestion of infected cells, and ORF7a might be used to distinguish macrophages based on their virally infected cell-ingestion status.

### 3.3. The Inferred Cellular-Trajectories Model the Cell Type-Specific Immune Response

**Figure 2:**
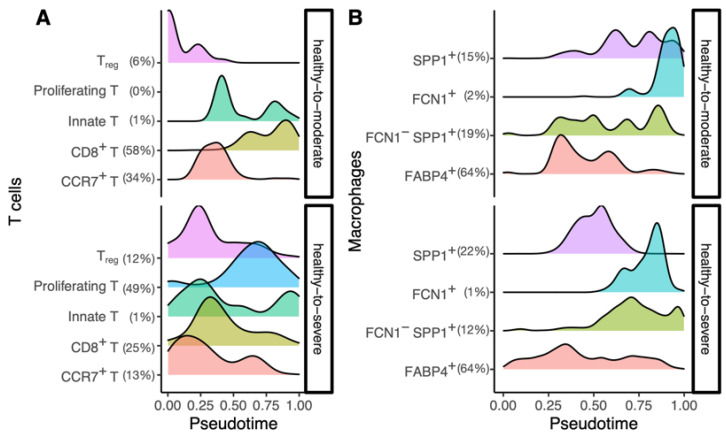
Changes of sub-cell type populations for each inferred cellular trajectory. (**A**) T cell sub-cell type changes of healthy-to-moderate (top) and healthy-to-severe (bottom). (**B**) Macrophage sub-cell type changes of healthy-to-moderate (top) and healthy-to-severe (bottom). The *x*-axis indicates the inferred pseudotime, and the *y*-axis indicates the height of density estimated and visualized by the geom_density function of ggplot2 R package [25]. A percentage next to a cell type name indicates the proportion of the cell type in the trajectory, and it is rounded to the ones place. Treg: regulatory T cells. CD8: cluster of differentiation 8. CCR7: C-C motif chemokine receptor 7. SPP1: secreted phosphoprotein 1. FCN1: Ficolin-1. FABP4: Fatty acid-binding protein 4.

Next, using the sub-cell type information of T cells and macrophages from Liao et al. [9], we observed the subtype population changes of each cellular trajectory throughout pseudotime to examine whether the trajectory could model the cell type-specific immune response. (Figure 2). We observed a proper immune response in the healthy-to-moderate trajectory of T cells, which ultimately resulted in an adaptive immune response by cytotoxic T cells (CD8^+^ T cells), resulting in infection clearance. In contrast, T cells’ healthy-to-severe trajectory showed the subpopulation responded early in the infection, at a similar time point, and decreased in numbers as the infection progressed (Figure 2A). Immune cell exhaustion has been observed in severe COVID-19 patients, and the T cell dynamics reflect it. In addition, macrophage subpopulations in the healthy-to-severe trajectory had an increased number of *FABP4*^+^ (a sub-cell type with high-expression of fatty acid-binding protein 4) and *FCN1*^-^*SPP1*^+^ (a sub-cell type with low-expression of ficolin-1 and high-expression of secreted phosphoprotein 1) throughout the trajectory (Figure 2B). This reflects the recruitment of proinflammatory monocytes towards the lungs, which has been observed in severe conditions. Our findings demonstrate that the inferred trajectories could model not only the COVID-19 progression but also the immune response.

### 3.4. DrivAER Identified Potential Transcriptional Programs That Differentiate The Severity of COVID-19

**Figure 3:**
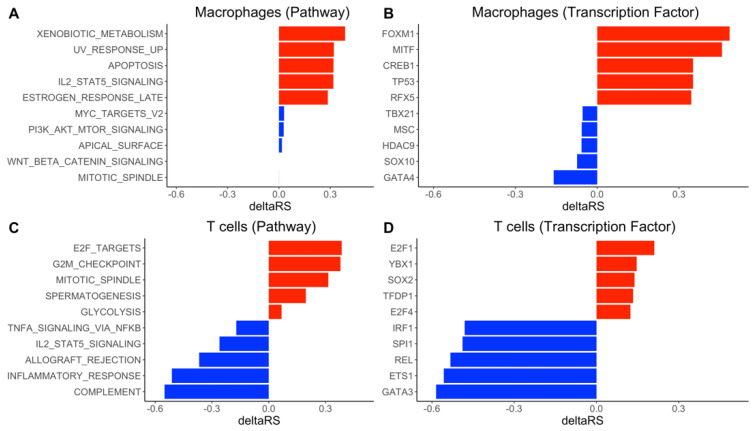
DrivAER analysis identified hallmark pathways and transcription factors differentially activated in macrophages and T cells. (**A**) The DrivAER pathway analysis results in macrophages. (**B**) The DrivAER transcription factor analysis results in macrophages. (**C**) The DrivAER pathway analysis results in T cells. (**D**) The DrivAER transcription factor analysis results in macrophages. Each bar indicates the ΔRS score (deltaRS) of the corresponding TP. The top five TPs (red bars) are the transcriptional programs that were highly expressed in the severe trajectory. The bottom five TPs (blue bars) are the top five transcriptional programs that were highly expressed in the moderate trajectory.

We performed transcriptional program analysis for the selected top lineage of each cellular trajectory using DrivAER (Figure 3 and Supplementary Table S2). To investigate whether SARS-CoV-2 genes directly regulate the trajectories, we first ran DrivAER using SARS-CoV-2 genes (only for H→S  in macrophages). We did not find a significant correlation between the SARS-CoV-2 gene expression and the cellular trajectory ($RS_{H\to S\;}=0.0019$, Supplementary Figure S3). This result suggested that the disease progression might not be directly regulated by the viral genes, rather it might be regulated by other biological mechanisms. 

**Figure 4:**
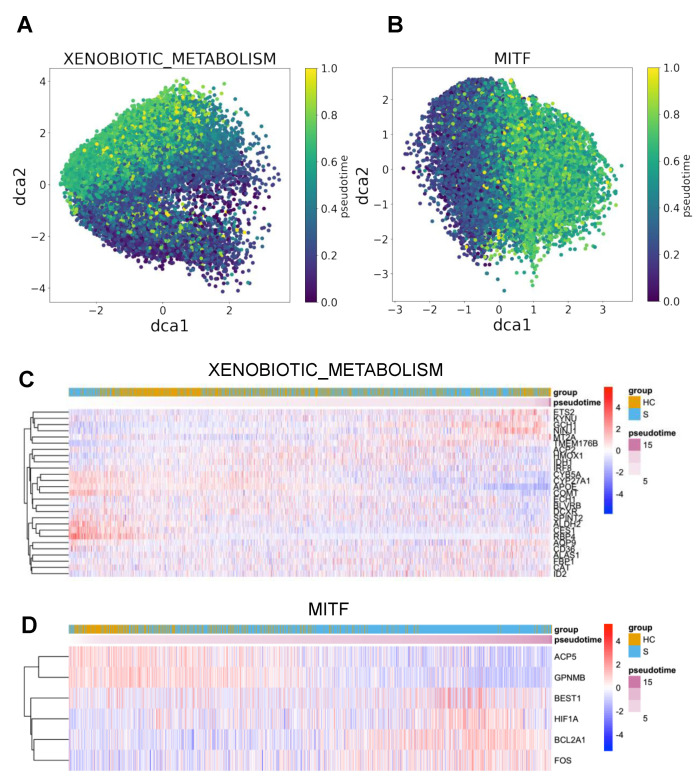
Visualization of both manifold and gene expression change of top transcriptional programs of the severe trajectory in macrophages. (**A**,**B**) A cell manifold of xenobiotic metabolism pathway and *MITF* transcription factor performed by DCA, respectively. X- and y-axes indicate the first and second dimensions of the gene manifold. Each point indicates a cell and is colored by its pseudotime. (**C**,**D**) Gene expression heatmaps for the top transcriptional programs (xenobiotic metabolism pathway and *MITF*). The transcriptional program genes expressed in less than 20% of cells, or the cells showing less than 40% of expressions of the transcriptional program’s genes were excluded during the heatmap visualization.

We next ran DrivAER using transcriptional program annotations from MSigDB [23] and TRRUST [24] and prioritized several hallmark pathways and TFs by their ΔRS values, which measure the differentiation of a selected TP between H→S and H→M. From the analysis of macrophages trajectories, we identified the xenobiotic metabolism pathway and the TF *MITF* (melanocyte inducing transcription factor) as the top transcriptional programs in healthy-to-severe pathogenesis on macrophages. The visualization of manifold gene expression of both top transcriptional programs showed marginally gradient patterns that follow their trajectory (Figure 4A,B), but both manifold gene expressions did not show any strong linear patterns that indicate strong correlations between the manifold gene expressions and pseudotimes. We also found that expressions of some genes in the transcriptional programs were moderately correlated with the inferred pseudotime (Figure 4C,D). We performed DrivAER on the sub-cell types of both T cells and macrophages (Supplementary Table S3). Proliferating T cells were not used during the analysis because the cell type’s healthy-to-moderate trajectory did not have enough number of cells. Our DrivAER analysis could not identify any transcriptional programs that showed stronger ΔRS for the sub-cell types than Macrophages or T cells (Supplementary Figure S4). 

## 4. Discussion

In this study, we utilized machine learning approaches to investigate the cellular trajectory in the severity of COVID-19. Using the slingshot algorithm, we first found that there were more diverse trajectories of H→S than H→M for both macrophages and T cells. Furthermore, our deep-learning algorithm DrivAER analysis found that the trajectories are not directly regulated by SARS-CoV-2 genes, but several transcriptional programs potentially drive the transcriptomic changes in COVID-19 pathogenesis and serve as the biomarkers of COVID-19 severity. 

We found several pieces of evidence from previous studies that the identified transcriptional programs could be keys to understanding the undiscovered mechanism of the pathogenesis of COVID-19 and differentiation between H→M and H→S. For example, the previous studies about the xenobiotic metabolism pathway hint that increased cytochrome P450 expression in macrophages worsens disease severity. The term xenobiotic refers to any chemical or substance that is exogenous to the system, specifically for humans in this case [26]. The cytochrome P450 protein family (CYPs) is the most important member of this pathway. The interaction between the immune system and CYPs during inflammation has previously been examined. Pro-inflammatory cytokines such as IL-6 and TNF-alpha have been shown to down-regulate CYP activity in the liver [27]. There are also CYPs expressed within alveolar lung macrophages [28]. However, our results show an increased activity of macrophage xenobiotic metabolism in patients with a severe symptom compared to healthy controls. The CYP activity has been reported to increase inflammation and inhibit macrophage phagocytic ability during sepsis [29]. This contributes to oxidative stress, which contributes to the cytokine storm observed in severe cases during COVID-19 infection [30]. We believe that initially, the SARS-CoV-2 virus triggers increased macrophage activity, and this increased xenobiotic metabolism reflects changes in an oxidative burst from macrophages after phagocytosis. In addition, as the infection progresses, the macrophages secrete various pro-inflammatory cytokines that recruit other pro-inflammatory monocytes to the infection site [31]. This creates a positive feedback loop that continues to increase the oxidative stress of the patient and ultimately creates a cytokine storm, resulting in significant immunopathology. 

We also found that dysregulation of TF MITF may result in severe COVID-19 infections. MITF is a transcription factor involved in the development of cell lineage, growth, and survival [32]. It was initially discovered in melanocytes and has also been studied in the context of melanoma [33]. In the context of the COVID-19 pandemic, Bost et al.’s study of SARS-CoV-2 host-viral infection maps identified *MITF* as one of the up-regulated genes [34]. MITF has been identified as a suppressor of innate immunity [35]. The gene for MITF lies downstream of M-CSF (macrophage colony-stimulating factor), a cytokine known as a growth factor for differentiation and growth of monocytes and macrophages [36]. The cytokine GM-CSF (granulocyte-macrophage colony-stimulating factor) has been explored as a potential therapy or therapeutic target for COVID-19 hyper-inflammation [37]. A randomized interventional trial suggested that administering recombinant GM-CSF improved patient outcomes, but there were questions that needed to be addressed as well [38]. Our results suggest that dysregulation of *MITF* in macrophages worsens infection severity in patients, but the mechanism behind this is not understood especially with the current evidence of macrophage-related inflammation in COVID-19 infections. 

In the T cell healthy-to-severe trajectory analysis, we found that increased T cell activity may be insufficient to control the severity of SARS-CoV-2 infection. The role of adaptive cell-mediated immunity, CD4^+^ and CD8^+^ T cells, in a viral infection, is well understood. T cell activity plays a major role in the clearance of a SARS-CoV-2 infection [39], and T cell dysfunction has been observed in the most severe COVID-19 cases [40,41]. Moreover, T cell overactivation and exhaustion contribute to the hyperinflammatory state observed in severe patients and enhance the immunopathology caused by the cytokine storm [42]. The G2M checkpoint pathway is involved in the cell cycle. It is where cells progress into mitosis unless DNA damage has occurred [43]. In addition, the TF E2F is also involved in the cell cycle by promoting cellular growth. Aberrant E2F pathway activation can result in inappropriate cellular entry into the S phase [44]. Our results indicate that T cells undergo increased cellular growth during a severe COVID-19 infection as seen in the ΔRS of the G2M checkpoint pathway and E2F pathway between healthy control and severe patients. We also observed increased mitosis as seen in the ΔRS of the mitotic spindle pathway between healthy control and severe patients [45]. This indicates that T cell growth and proliferation are still functional but insufficient to control the virus in a severe COVID-19 infection.

We also observed the preservation of complement pathway activation in the H→M trajectory of T cells (Figure 3C). The complement cascade is another arm of the innate immune system. Proteins involved in the cascade can initiate opsonization by phagocytes or directly attack pathogens by forming a membrane attack complex [46]. In addition, complement acts as a link between the innate and adaptive immune systems to allow for a coordinated response during infection [47]. Complement’s role in COVID-19 infection has been highlighted recently as well. Complement dysfunction has been associated with respiratory failure [48]. It also comprises one of the multiple factors associated with severe infections [49]. Damage from complement dysfunction has been observed in autoimmune infections where complement cascade targets the host’s cells [50]. The complement system may worsen inflammation in disease as well [51]. As stated earlier, the hyperinflammatory state during a COVID-19 infection is a major factor in a severe infection. Complement increases the cell response via increased IFN-gamma activity [52,53]. IFN-gamma is classified as a pro-inflammatory cytokine [54]. Our results may support that preserved complement cascade in T cells results in a more favorable outcome for patients infected by SARS-CoV-2. 

It is worth noting that the inflammatory response pathway appears to be stronger in H→M than H→S on T cells (Figure 3C). However, our results did not actually indicate that moderate patients exhibit a greater inflammatory response than the severe patients. We observed that the severe patients exhibited a higher level of gene expression of inflammatory response pathway genes (Supplementary Figure S5), and we suspected the higher expression might indicate their inflammatory response signature to remain constant throughout the infection.

The transcription factor analysis of H→M trajectory of T cells showed that activation of GATA3 coincides with decreased cytokine secretion and a better infection outcome. GATA3 holds an important role in the development and function of Th2 cells [55]. GATA3 also contributes to the ability of Th2 cells to secrete cytokines such as IL-4, 5, and 13, which are required for a type 2 immune response [56]. The type-2 immune response is characterized as immunity against helminths and parasites [57]. However, it is the type-1 response that is required to fight against intracellular pathogens including SARS-CoV-2. The type-2 immune response has also been shown to negatively impact the course of infections by respiratory viruses due to their promotion of inflammatory cells such as eosinophils [58]. The *GATA3* gene is highly expressed in T cells of moderate patients (Wilcox signed-rank test, *p*-value < 2.2 × 10^−16^, while it is less expressed in T cells of severe patients. This validates our results as GATA3 activity should be down-regulated during a viral infection. These patients with a severe infection may have an active Th1 response as a result of GATA3 downregulation. On the other hand, moderate patients expressing a higher level of GATA3 may be a result of a lighter infection load. It is also possible there is some unknown mechanism causing this as cytokine profiles of severe COVID-19 patients are skewed towards a Th2 response [59]. 

We acknowledge the limitations in our studies including but not limited to the small sample size and lack of experimental validation. BALF is normally collected during the bronchoalveolar washing, which is a diagnostic tool for uncommon condition in lower respiratory tract pathology. This would greatly limit the scale of the sample collection. Moreover, after we checked all the available COVID-19 BALF single-cell datasets, we found there are no other studies that have a comparable study design, severity definition, or sample size. Furthermore, due to the high infectivity and pathogenicity of SARS-CoV-2, only a limited number of labs would have the capability to conduct functional validations. This is a common problem in the COVID-19 research field. Thus, further validation for our results is warranted when additional data is released. Other limitations include the type of data required for this analysis. Our dataset did not contain any developmental data and the number of patients was low, possibly resulting in a lower statistical power than desired. The cellular trajectory is also one-directional and does not consider more complex cases. In the future, we believe this method could be applied to other datasets and diseases especially in diseases where patients exhibit a great deal of heterogeneity.

## 5. Conclusions

We used machine learning approaches to investigate the cellular status transition of macrophages and T cells in diverse COVID-19 severity. We identified macrophage-related functions (xenobiotic metabolism pathway and binding of MITF) that contribute more to the severe COVID-19 symptoms. On the other hand, the deficiency of certain T cell-related functions (complement pathway and binding of GATA3) will likely lead to severe infection. Our findings provide new insight into the disease pathogenesis and potential treatment of COVID-19.

## Figures and Tables

**Table 1:** The summary of top inferred cellular trajectories of macrophages and T cells.

Cell Type	Trajectory	Number of Healthy Cells	Number of Moderate/Severe Cells	Fold-Change	*p*-Value
Macrophage	H→M	6370	2168	0.37	$9.52\times 10^{-142}$
	H→S	10,492	19,798	0.94	$0\times 10^{0}$
T cell	H→M	414	1524	0.47	$3.66\times 10^{-27}$
	H→S	237	975	0.56	$1.74\times 10^{-20}$

**Table 2:** The statistics of the SARS-nCoV-2 gene expression across the pseudotime.

Cell Type	Trajectory	Gene	Number of Infected Cells	Average (Pseudotime)
Macrophage	H→S	S	104	0.46 ± 0.19
		ORF8	41	0.46 ± 0.20
		N	258	0.48 ± 0.21
		ORF10	67	0.49 ± 0.20
		ORF3a	24	0.49 ± 0.21
		M	34	0.50 ± 0.22
		ORF1ab	467	0.52 ± 0.21
		ORF7a	403	0.57 ± 0.18
T cell	H→S	ORF1ab	38	0.61 ± 0.27

## Data Availability

The raw scRNA-seq files supporting the conclusions of this article can be downloaded from the NCBI GEO database (GEO ID: GSE145926), and the pre-processed gene-expression data are available at https://covid19-balf.cells.ucsc.edu.

## Supplementary Materials

The following are available online at https://www.mdpi.com/article/10.3390/genes12050635/s1, Figure S1: The illustrations of UMAP embedded cells (colored by the disease status) and inferred cellular trajectories (displayed lines). Bold curved lines represent the selected top trajectories for the downstream analysis. H→M trajectories on macrophages and T cells (top). H→S trajectories on macrophages and T cells (bottom). Figure S2: SARS-CoV-2 gene expressions across cellular trajectories between healthy (H) and severe (S) patients in macrophages and T cells. Each row indicates a SAR-CoV-2 gene, each boxplot indicates the distribution of the SAR-CoV-2 gene expression, and a dot on a box plot indicates a cell that shows the gene expression at the pseudotime. (A) SARS-CoV-2 gene-expression in macrophages. (B) SARS-CoV-2 gene expressions in T cells. Figure S3: The SARS-CoV-2 gene manifold of the *H*→*S* trajectory of macrophages. The gene manifold was performed by DCA. X- and y-axes indicate the first and second dimension of the gene manifold. A point indicates a cell, and its color indicates the cell’s pseudotime. Figure S4: ΔRS distributions for each cell type. Figure S5: average expressions and percentages of expressing cells of inflammatory response pathway genes by disease status. Table S1: The summary of inferred cellular trajectories and their lineages on macrophages and T-cells. Selected lineages of each trajectory are marked by an asterisk. Table S2: The list of TPs, relevance scores ($RS_{H\to M}$ and $\;RS_{H\to S}$), and ΔRS obtained by DrivAER analysis on macrophages and T cells. Table S3: The list of TPs, relevance scores ($RS_{H\to M}$ and $\;RS_{H\to S}$), and ΔRS obtained by DrivAER analysis on sub-cell types.

## References

[1] CDC COVID Data Tracker. https://covid.cdc.gov/covid-data-tracker/.

[2] Ahmed A., Ali A., Hasan S. (2020). Comparison of Epidemiological Variations in COVID-19 Patients Inside and Outside of China-A Meta-Analysis. Front. Public Health.

[3] Goodman K.E., Magder L.S., Baghdadi J.D., Pineles L., Levine A.R., Perencevich E.N., Harris A.D. (2020). Impact of Sex and Metabolic Comorbidities on COVID-19 Mortality Risk Across Age Groups: 66,646 Inpatients Across 613 U.S. Hospitals. Clin. Infect. Dis..

[4] Huang C., Wang Y., Li X., Ren L., Zhao J., Hu Y., Zhang L., Fan G., Xu J., Gu X. (2020). Clinical features of patients infected with 2019 novel coronavirus in Wuhan, China. Lancet.

[5] Xiong J., Lipsitz O., Nasri F., Lui L.M.W., Gill H., Phan L., Chen-Li D., Iacobucci M., Ho R., Majeed A. (2020). Impact of COVID-19 pandemic on mental health in the general population: A systematic review. J. Affect. Disord..

[6] Gustine J.N., Jones D. (2021). Immunopathology of Hyperinflammation in COVID-19. Am. J. Pathol..

[7] Ye Q., Wang B., Mao J. (2020). The pathogenesis and treatment of the `Cytokine Storm’ in COVID-19. J. Infect..

[8] Dai Y., Hu R., Manuel A.M., Liu A., Jia P., Zhao Z. (2021). CSEA-DB: An omnibus for human complex trait and cell type associations. Nucleic Acids Res..

[9] Liao M., Liu Y., Yuan J., Wen Y., Xu G., Zhao J., Cheng L., Li J., Wang X., Wang F. (2020). Single-cell landscape of bronchoalveolar immune cells in patients with COVID-19. Nat. Med..

[10] Liu T., Jia P., Fang B., Zhao Z. (2020). Differential Expression of Viral Transcripts From Single-Cell RNA Sequencing of Moderate and Severe COVID-19 Patients and Its Implications for Case Severity. Front. Microbiol..

[11] Xu G., Qi F., Li H., Yang Q., Wang H., Wang X., Liu X., Zhao J., Liao X., Liu Y. (2020). The differential immune responses to COVID-19 in peripheral and lung revealed by single-cell RNA sequencing. Cell Discov..

[12] Zhao Z., Li H., Wu X., Zhong Y., Zhang K., Zhang Y.-P., Boerwinkle E., Fu Y.-X. (2004). Moderate mutation rate in the SARS coronavirus genome and its implications. BMC Evol. Biol..

[13] Liu S., Shen J., Fang S., Li K., Liu J., Yang L., Hu C.-D., Wan J. (2020). Genetic spectrum and distinct evolution patterns of SARS-CoV-2. Front. Microbiol..

[14] Street K., Risso D., Fletcher R.B., Das D., Ngai J., Yosef N., Purdom E., Dudoit S. (2018). Slingshot: Cell lineage and pseudotime inference for single-cell transcriptomics. BMC Genom..

[15] Simon L.M., Yan F., Zhao Z. (2020). DrivAER: Identification of driving transcriptional programs in single-cell RNA sequencing data. Gigascience.

[16] UCSC Cell Browser. https://cells.ucsc.edu/?ds=covid19-balf.

[17] McInnes L., Healy J., Melville J. (2018). *UMAP:* Uniform Manifold Approximation and Projection for Dimension Reduction. arXiv.

[18] Stuart T., Butler A., Hoffman P., Hafemeister C., Papalexi E., Mauck W.M., Hao Y., Stoeckius M., Smibert P., Satija R. (2019). Comprehensive Integration of Single-Cell Data. Cell.

[19] Fu J., Akat K.M., Sun Z., Zhang W., Schlondorff D., Liu Z., Tuschl T., Lee K., He J.C. (2019). Single-Cell RNA Profiling of Glomerular Cells Shows Dynamic Changes in Experimental Diabetic Kidney Disease. J. Am. Soc. Nephrol..

[20] Saelens W., Cannoodt R., Todorov H., Saeys Y. (2019). A comparison of single-cell trajectory inference methods. Nat. Biotechnol..

[21] Heimberg G., Bhatnagar R., El-Samad H., Thomson M. (2016). Low Dimensionality in Gene Expression Data Enables the Accurate Extraction of Transcriptional Programs from Shallow Sequencing. Cell Syst..

[22] Eraslan G., Simon L.M., Mircea M., Mueller N.S., Theis F.J. (2019). Single-cell RNA-seq denoising using a deep count autoencoder. Nat. Commun..

[23] Liberzon A., Birger C., Thorvaldsdóttir H., Ghandi M., Mesirov J.P., Tamayo P. (2015). The Molecular Signatures Database (MSigDB) hallmark gene set collection. Cell Syst..

[24] Han H., Cho J.-W., Lee S., Yun A., Kim H., Bae D., Yang S., Kim C.Y., Lee M., Kim E. (2018). TRRUST v2: An expanded reference database of human and mouse transcriptional regulatory interactions. Nucleic Acids Res..

[25] Wickham H. (2011). ggplot2. WIRes Comput. Stat..

[26] Patterson A.D., Gonzalez F.J., Idle J.R. (2010). Xenobiotic metabolism: A view through the metabolometer. Chem. Res. Toxicol..

[27] El-Ghiaty M.A., Shoieb S.M., El-Kadi A.O.S. (2020). Cytochrome P450-mediated drug interactions in COVID-19 patients: Current findings and possible mechanisms. Med. Hypotheses.

[28] Hukkanen J., Pelkonen O., Raunio H. (2001). Expression of xenobiotic-metabolizing enzymes in human pulmonary tissue: Possible role in susceptibility for ILD. Eur. Respir. J. Suppl..

[29] Tian L.-X., Tang X., Zhu J.-Y., Luo L., Ma X.-Y., Cheng S.-W., Zhang W., Tang W.-Q., Ma W., Yang X. (2020). Cytochrome P450 1A1 enhances inflammatory responses and impedes phagocytosis of bacteria in macrophages during sepsis. Cell Commun. Signal..

[30] Cecchini R., Cecchini A.L. (2020). SARS-CoV-2 infection pathogenesis is related to oxidative stress as a response to aggression. Med. Hypotheses.

[31] Otsuka R., Seino K.-I. (2020). Macrophage activation syndrome and COVID-19. Inflamm. Regen..

[32] Kawakami A., Fisher D.E. (2017). The master role of microphthalmia-associated transcription factor in melanocyte and melanoma biology. Lab. Investig..

[33] Garraway L.A., Sellers W.R. (2006). Lineage dependency and lineage-survival oncogenes in human cancer. Nat. Rev. Cancer.

[34] Bost P., Giladi A., Liu Y., Bendjelal Y., Xu G., David E., Blecher-Gonen R., Cohen M., Medaglia C., Li H. (2020). Host-Viral Infection Maps Reveal Signatures of Severe COVID-19 Patients. Cell.

[35] Harris M.L., Fufa T.D., Palmer J.W., Joshi S.S., Larson D.M., Incao A., Gildea D.E., Trivedi N.S., Lee A.N., Day C.-P. (2018). A direct link between MITF, innate immunity, and hair graying. PLoS Biol..

[36] Douglass T.G., Driggers L., Zhang J.G., Hoa N., Delgado C., Williams C.C., Dan Q., Sanchez R., Jeffes E.W.B., Wepsic H.T. (2008). Macrophage colony stimulating factor: Not just for macrophages anymore! A gateway into complex biologies. Int. Immunopharmacol..

[37] Mehta P., Porter J.C., Manson J.J., Isaacs J.D., Openshaw P.J.M., McInnes I.B., Summers C., Chambers R.C. (2020). Therapeutic blockade of granulocyte macrophage colony-stimulating factor in COVID-19-associated hyperinflammation: Challenges and opportunities. Lancet Respir. Med..

[38] Cheng L.-L., Guan W.-J., Duan C.-Y., Zhang N.-F., Lei C.-L., Hu Y., Chen A.-L., Li S.-Y., Zhuo C., Deng X.-L. (2021). Effect of Recombinant Human Granulocyte Colony-Stimulating Factor for Patients With Coronavirus Disease 2019 (COVID-19) and Lymphopenia: A Randomized Clinical Trial. JAMA Intern. Med..

[39] de Candia P., Prattichizzo F., Garavelli S., Matarese G. (2021). T Cells: Warriors of SARS-CoV-2 Infection. Trends Immunol..

[40] Qin C., Zhou L., Hu Z., Zhang S., Yang S., Tao Y., Xie C., Ma K., Shang K., Wang W. (2020). Dysregulation of Immune Response in Patients With Coronavirus 2019 (COVID-19) in Wuhan, China. Clin. Infect. Dis..

[41] Dai Y., Wang J., Jeong H.-H., Chen W., Jia P., Zhao Z. (2021). Association of CXCR6 with COVID-19 severity: Delineating the host genetic factors in transcriptomic regulation. bioRxiv.

[42] Liu L., Xu L., Lin C. (2020). T cell response in patients with COVID-19. Blood Sci..

[43] Stark G.R., Taylor W.R. (2004). Analyzing the G2/M checkpoint. Methods Mol. Biol..

[44] Kirkham P.A., Lam E.W., Takamatsu H.H., Parkhouse R.M. (1998). Transcription factor E2F controls the reversible gamma delta T cell growth arrest mediated through WC1. J. Immunol..

[45] Inoué S. (1981). Cell division and the mitotic spindle. J. Cell Biol..

[46] Janeway C.A., Travers P., Walport M., Shlomchik M.J. (2001). The Complement System and Innate Immunity.

[47] Dunkelberger J.R., Song W.-C. (2010). Complement and its role in innate and adaptive immune responses. Cell Res..

[48] Holter J.C., Pischke S.E., de Boer E., Lind A., Jenum S., Holten A.R., Tonby K., Barratt-Due A., Sokolova M., Schjalm C. (2020). Systemic complement activation is associated with respiratory failure in COVID-19 hospitalized patients. Proc. Natl. Acad. Sci. USA.

[49] Java A., Apicelli A.J., Liszewski M.K., Coler-Reilly A., Atkinson J.P., Kim A.H., Kulkarni H.S. (2020). The complement system in COVID-19: Friend and foe?. JCI Insight.

[50] Defendi F., Thielens N.M., Clavarino G., Cesbron J.-Y., Dumestre-Pérard C. (2020). The Immunopathology of Complement Proteins and Innate Immunity in Autoimmune Disease. Clin. Rev. Allergy Immunol..

[51] Markiewski M.M., Lambris J.D. (2007). The role of complement in inflammatory diseases from behind the scenes into the spotlight. Am. J. Pathol..

[52] Kwan W.-H., van der Touw W., Heeger P.S. (2012). Complement regulation of T cell immunity. Immunol. Res..

[53] Peng W., McKenzie J.A., Hwu P. (2016). Complementing T-cell Function: An Inhibitory Role of the Complement System in T-cell-Mediated Antitumor Immunity. Cancer Discov..

[54] Lee S.H., Kwon J.Y., Kim S.-Y., Jung K., Cho M.-L. (2017). Interferon-gamma regulates inflammatory cell death by targeting necroptosis in experimental autoimmune arthritis. Sci. Rep..

[55] Sasaki T., Onodera A., Hosokawa H., Watanabe Y., Horiuchi S., Yamashita J., Tanaka H., Ogawa Y., Suzuki Y., Nakayama T. (2013). Genome-Wide Gene Expression Profiling Revealed a Critical Role for GATA3 in the Maintenance of the Th2 Cell Identity. PLoS ONE.

[56] Tindemans I., Serafini N., Di Santo J.P., Hendriks R.W. (2014). GATA-3 function in innate and adaptive immunity. Immunity.

[57] Koyasu S., Moro K. (2011). Type 2 innate immune responses and the natural helper cell. Immunology.

[58] Roncati L., Nasillo V., Lusenti B., Riva G. (2020). Signals of Th2 immune response from COVID-19 patients requiring intensive care. Ann. Hematol..

[59] Li C.K.-F., Wu H., Yan H., Ma S., Wang L., Zhang M., Tang X., Temperton N.J., Weiss R.A., Brenchley J.M. (2008). T cell responses to whole SARS coronavirus in humans. J. Immunol..

