# Supplementary figures and images for: Investigating Cellular Trajectories in the Severity of COVID-19 and Their Transcriptional Programs Using Machine Learning Approaches
Source: Genes (Basel). 2021 Apr 24;12(5):635. doi: 10.3390/genes12050635 (PMC8145325; doi:10.3390/genes12050635)

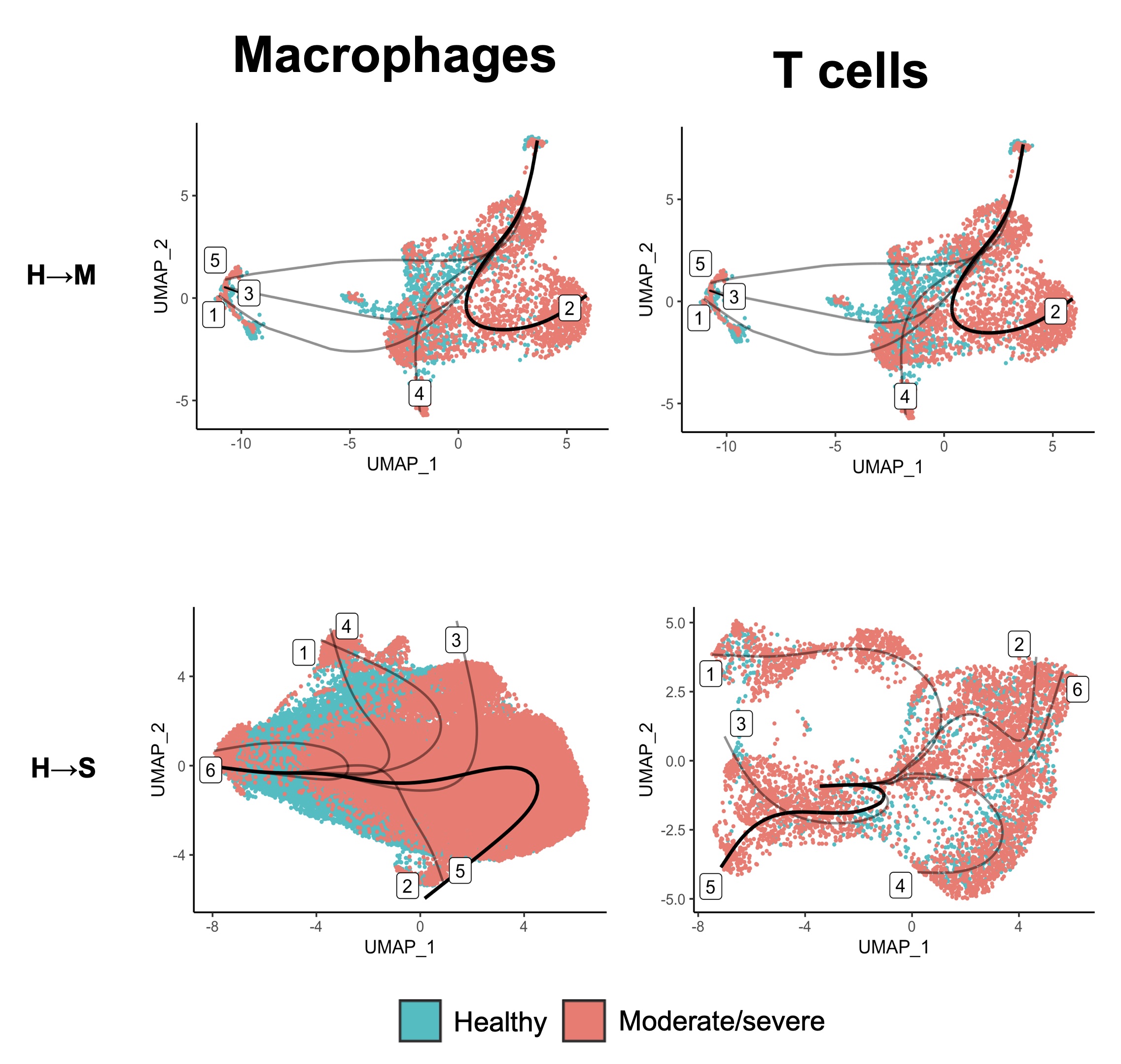

Supplement: Supplementary file 1 [file genes-12-00635-s001.zip › Supplementary Materials/Figure_S1.jpg]

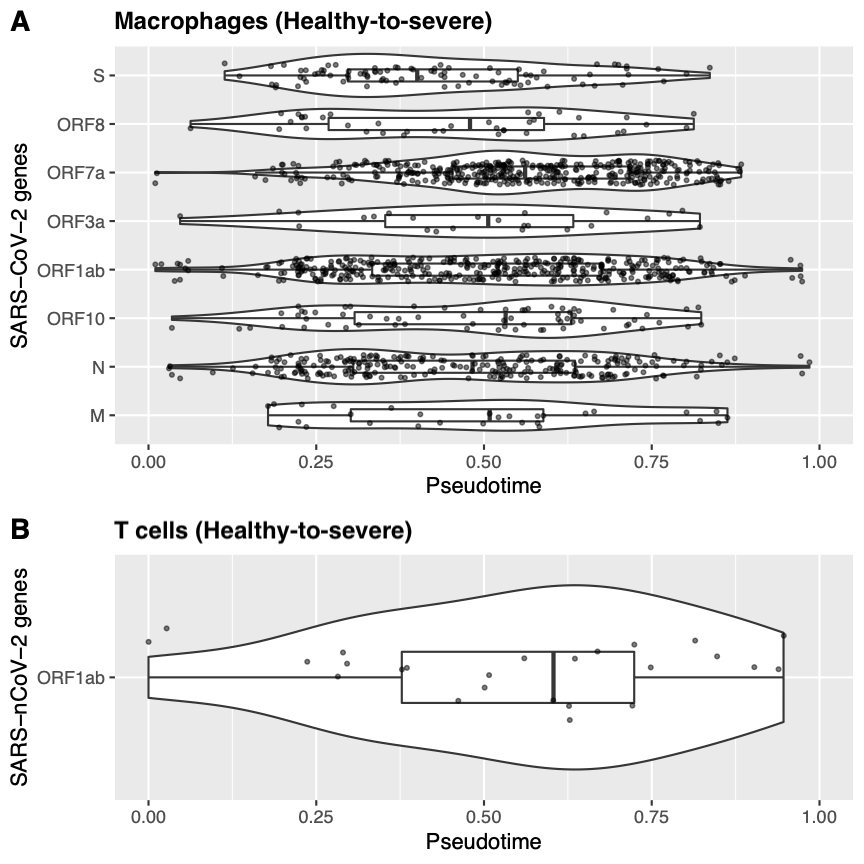

Supplement: Supplementary file 1 [file genes-12-00635-s001.zip › Supplementary Materials/Figure_S2.jpg]

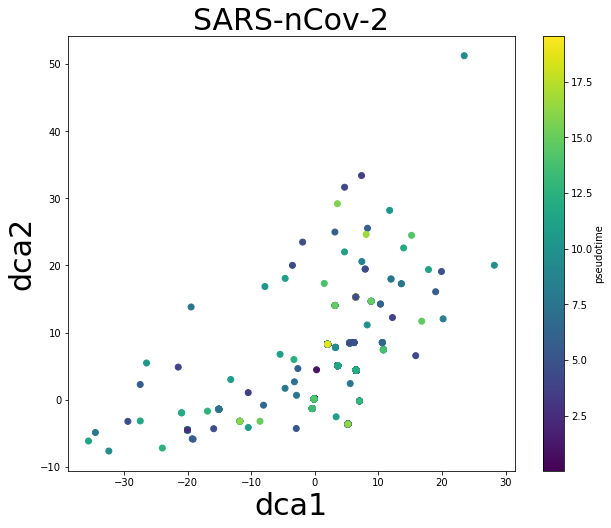

Supplement: Supplementary file 1 [file genes-12-00635-s001.zip › Supplementary Materials/Figure_S3.png]

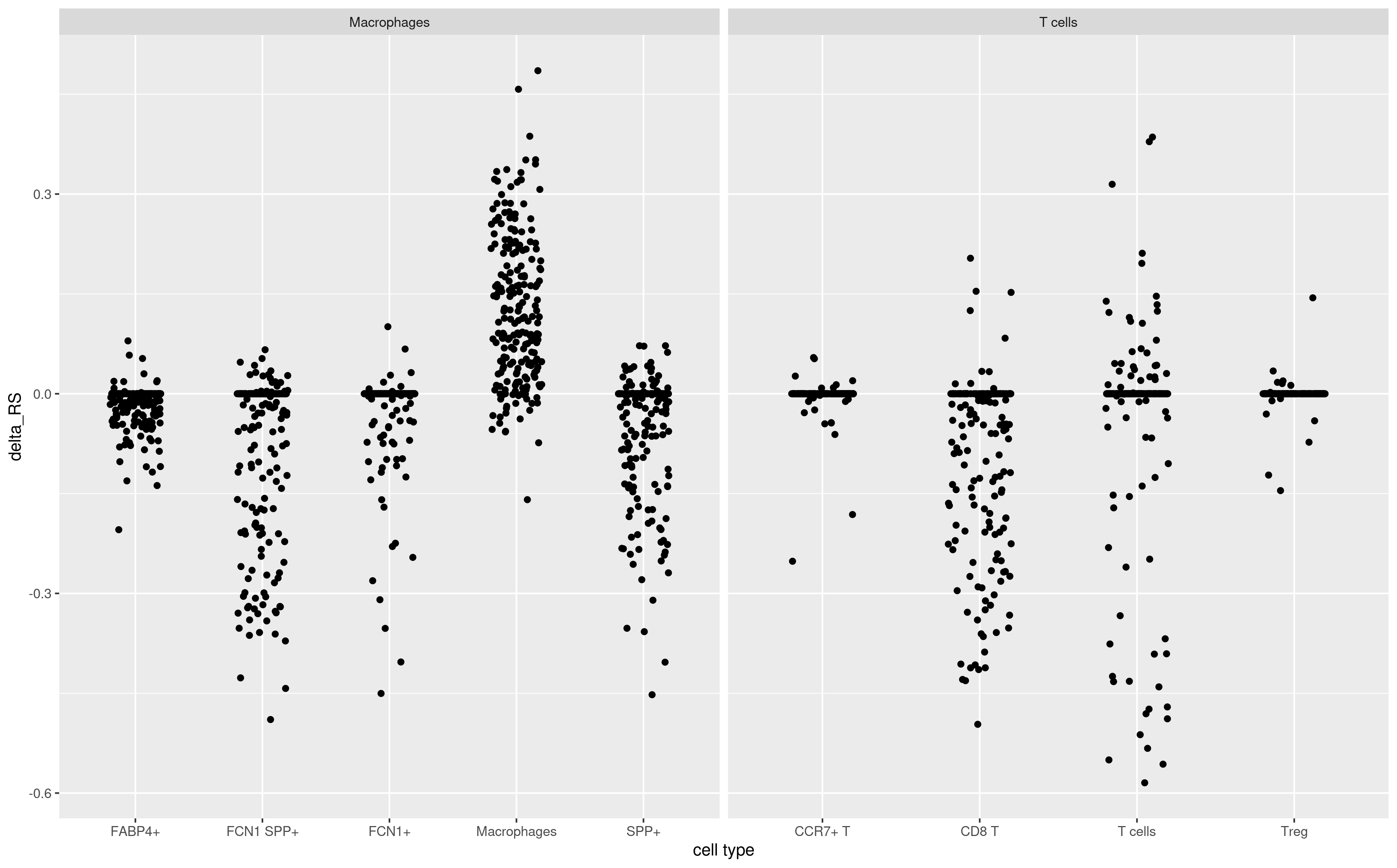

Supplement: Supplementary file 1 [file genes-12-00635-s001.zip › Supplementary Materials/Figure_S4.png]

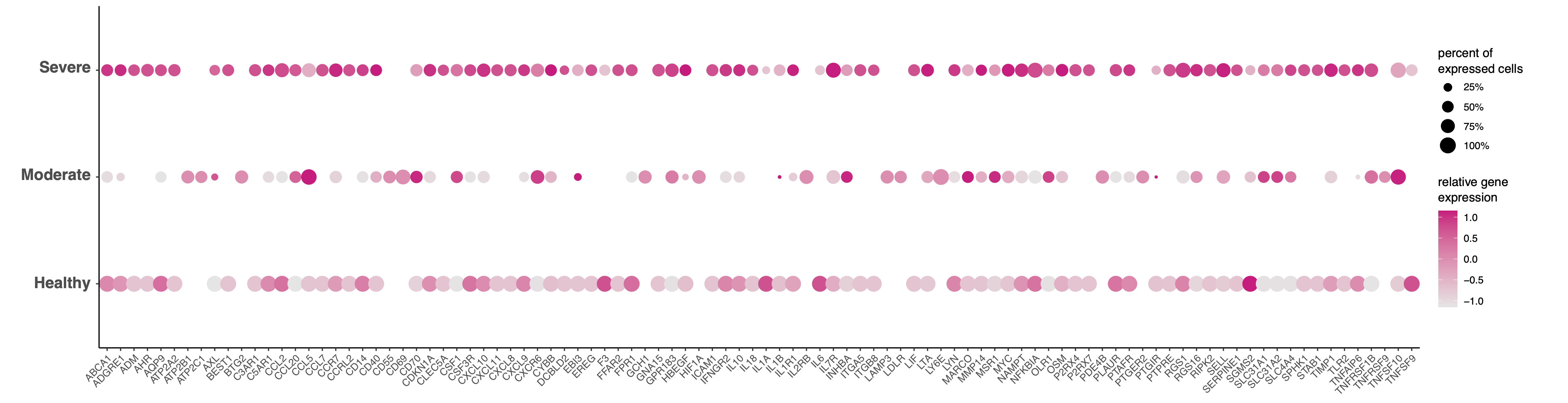

Supplement: Supplementary file 1 [file genes-12-00635-s001.zip › Supplementary Materials/Figure_S5.png]
